# Draft Genome Sequences of* Alloscardovia macacae* UMA81211 and UMA81212, Isolated from the Feces of a Rhesus Macaque (*Macaca mulatta*)

**DOI:** 10.1128/genomeA.00581-17

**Published:** 2017-06-29

**Authors:** Korin Albert, David A. Sela

**Affiliations:** a Department of Food Science, University of Massachusetts, Amherst, Massachusetts, USA; b Molecular and Cellular Biology Graduate Program, University of Massachusetts, Amherst, Massachusetts, USA; c Department of Microbiology, University of Massachusetts, Amherst, Massachusetts, USA; d Department of Microbiology and Physiological Systems, University of Massachusetts Medical School, Worcester, Massachusetts, USA

## Abstract

Here, we provide the draft genome sequences of two isolates identified as *Alloscardovia macacae*. These bacteria originated from the feces of a rhesus macaque. The draft genomes of both *Alloscardovia macacae* isolates are ~1.8 Mb in length, with a G+C content of 56.1%.

## GENOME ANNOUNCEMENT

In order to study the microbial communities that colonize the rhesus macaque (*Macaca mulatta*) gastrointestinal tract, fresh fecal samples were collected for analysis. Fecal samples were mixed with 5 ml of sterile peptone water and spread onto bifidobacterium-specific medium (BSM) agar, which consists of 55 g/liter de Man-Rogosa-Sharpe (MRS) agar (Difco), 15 g/liter agar, 0.05% (wt/vol) l-cysteine (Sigma-Aldrich), and 0.05% (wt/vol) mupirocin (AppliChem Panreac). Agar plates were incubated at 37°C for 24 h in a Coy anaerobic chamber. Individual colonies were picked and grown in BSM broth for 12 h under the same conditions. Liquid cultures were preserved as freezer stocks at -80°C using a 25% glycerol solution.

Genomic DNA was extracted using the MasterPure Gram-positive DNA purification kit and then further processed using the Genomic DNA Clean & Concentrator (Zymo Research). Sequencing libraries were prepared with the Nextera XT 150-bp paired-end library preparation kit (Illumina). Subsequently, whole-genome sequencing of the isolates was performed on an Illumina NextSeq using the version 2 reagent kit. Reads were assembled *de novo* using the SPAdes genome assembler 3.9.1 (1). Gene model prediction and annotation was performed using RAST (2).

The sequences of the 16S rRNA marker genes were obtained from the RAST annotations of UMA81211 and UMA81212 and showed 98.67% and 98.27% similarity to *Alloscardovia macacae* M8, respectively (3, 4). The next highest similarity scores were for *Alloscardovia criceti* DSM17774 (UMA81211, 96.54%; UMA81212, 96.31%) and *Alloscardovia omnicolens* DSM21503 (UMA81211, 96.54%; UMA81212, 95.93%) (3, 5). The full 16S rRNA sequences of UMA81211, UMA81212, and representative members of the family *Bifidobacteriaceae* were used to generate a neighbor-joining phylogenetic tree with bootstrapping using MEGA7 and the EZBioCloud database (4, 6).

### Accession number(s).

The genome sequences have been made publicly available with GenBank accession numbers NEKB00000000 and NEKC00000000.
